# Cerebral Tissue Oxygen Saturation Correlates with Emergence from Propofol-Remifentanil Anesthesia: An Observational Cohort Study

**DOI:** 10.3390/jcm11164878

**Published:** 2022-08-19

**Authors:** Jianxi Zhang, Zhigang Cheng, Ying Tian, Lili Weng, Yiying Zhang, Xin Yang, Michael K. E. Schäfer, Qulian Guo, Changsheng Huang

**Affiliations:** 1 Department of Anesthesiology, Xiangya Hospital Central South University, Changsha 410008, China; 2 National Clinical Research Center for Geriatric Disorders, Xiangya Hospital Central South University, Changsha 410008, China; 3 Department of Anesthesiology, University Medical Center, Johannes Gutenberg-University Mainz, 55122 Mainz, Germany; 4 Focus Program Translational Neurosciences (FTN), Research Center of Immunotherapy, Johannes Gutenberg-University Mainz, 55122 Mainz, Germany

**Keywords:** general anesthesia, cerebral tissue oxygen saturation, near-infrared spectroscopy, emergence

## Abstract

Anesthesia emergence is accompanied by changes in cerebral circulation. It is unknown whether cerebral tissue oxygen saturation (SctO_2_) could be an indicator of emergence. Changes in SctO_2_, bispectral index (BIS), mean arterial pressure (MAP), and heart rate (HR) were evaluated during the emergence from propofol-remifentanil anesthesia. At the time of cessation of anesthetic delivery, SctO_2_, BIS, MAP, and HR values were recorded as baseline. The changes of these parameters from the baseline were recorded as Δ SctO_2_, Δ BIS, Δ MAP, and Δ HR. The behavioral signs (body movement, coughing, or eye opening) and response to commands (indicating regaining of consciousness) were used to define emergence states. Prediction probability (Pk) was used to examine the accuracy of SctO_2_, BIS, MAP, and HR as indicators of emergence. SctO_2_ showed an abrupt and distinctive increase when appearing behavioral signs. BIS, MAP, and HR, also increased but with a large inter-individual variability. Pk value of Δ SctO_2_ was 0.97 to predict the appearance behavioral signs from 2 min before that, which was much higher than the Pk values of Δ BIS (0.81), Δ MAP (0.71) and Δ HR (0.87). The regaining of consciousness was associated with a further increase in the SctO_2_ value.

## 1. Introduction

Emergence from anesthesia is the final stage of anesthesia with the transition from unconsciousness to wakefulness. Rapid and accurate identification of the emergence state is critical for patient safety and reducing the risk of anesthesia. In clinical practice, anesthesiologists conventionally assess the level of arousal based on the interpretation of clinical signs and symptoms [1]. However, the different experience and the subjectivity of the practitioners could bias the interpretation. In addition, medical conditions, such as motor dysfunction or psychiatric disorders, can also confuse decision-making based on clinical assessment [2,3]. A combination with objective techniques that indicate the state of arousal is therefore essential for a better control of anesthesia emergence and patient’s wellbeing.

Currently, electroencephalogram (EEG)-derived brain monitors, such as Bispectral Index (BIS), SEDLine, entropy, narcotrend, and auditory evoked potential (AEP) are used to measure the anesthesia and emergence states [4,5,6,7]. Intraoperatively, monitoring of EEG response has been shown to improve the ability of anesthesiologists to titrate anesthetic drugs and reduce the risk of awareness [8,9]. However, these monitoring systems have limitations when used to indicate the emergence from anesthesia [10]. First, EEG-based algorithms are poor at tracking rapid changes during emergence. BIS and AEP index have weak predictive power with respect to movement in response to noxious stimuli [11]. BIS and entropy showed wide inter-individual variability and thus did not reliably differentiate consciousness from unconsciousness [12]. Second, these EEG monitors do not reflect the hypnotic state consistently. Tiefenthaler et al. [13] have shown that only 20% of BIS, AEP index and entropy values simultaneously categorized the state of anesthesia and wakefulness.

The anesthesia emergence is associated with increased neural activities [14,15], increased cerebral metabolic rate of oxygen (CMRO_2_), and increased cerebral blood flow (CBF) [16,17,18]. Currently, there are no clinical monitors that directly assess CMRO_2_ and CBF. Instead, the CMRO_2_-CBF balance can be monitored using cerebral oximetry based on near-infrared spectroscopy [19,20]. No studies have reported the change on cerebral tissue oxygen saturation (SctO_2_) during emergence.

Neuronal activation alters the CMRO_2_-CBF balance as it typically leads to a more pronounced increase in the CBF than in the CMRO_2_ due to cerebral coupling [21,22,23]. Previous studies reported that the concentration of deoxyhemoglobin was reduced during emergence from general anesthesia [24,25], indicating that cerebral oxygen supply may exceed the oxygen extraction. Therefore, in this observational cohort study, we hypothesize that SctO_2_ increases during anesthesia emergence. Our aim was to compare the pattern of SctO_2_ change with that of BIS change during emergence from propofol-remifentanil anesthesia, and to evaluate whether SctO_2_ could be an objective indicator of anesthesia emergence.

## 2. Methods

### 2.1. Study Design and Setting

This is an observational cohort study, which was conducted at Xiangya Hospital of Central South University from 15 April 2019 to 10 January 2020. All procedures of this study were approved by the Ethics Committee of Xiangya Hospital of Central South University (IRB No.201904111) and written informed consent was obtained from all subjects participating in the trial. The trial was registered prior to patient enrollment on the Chinese Clinical Trial Registry (Ref: ChiCTR1900021122, Principal investigator: Changsheng Huang, Date of registration: 29 January 2019). The work has been reported in line with the STROCSS criteria [26].

### 2.2. Participants

Patients who (1) were going to undergo general anesthesia and patients whose (2) age were 18 yr or older, and (3) ASA classification ranged from I to III were included. Patients who (1) had severe intraoperative organ failure requiring rescue, (2) were going to undergo craniocerebral surgery, (3) were unwilling to participate in the study or had participated in other clinical studies, (4) comorbid with serious diseases, and had a history of central nervous system diseases, cerebrovascular disease, cognitive impairment, mental disorders, and communication disorders were excluded. During the study, participants who had (1) postoperative agitation, (2) postoperative hypoxemia, (3) a deficiency of data and (4) medications that may affect the results (sedatives, central stimulants, etc.) after cessation of anesthetic delivery were eliminated.

### 2.3. Study Procedures

Anesthesia monitors were applied prior to the start of anesthetic delivery. The monitors included noninvasive blood pressure, electrocardiogram, pulse oximetric oxygen saturation (SpO_2_), body temperature, BIS and SctO_2_. The BIS VISTA monitor (Aspect Medical Systems, Newton, MA, USA) was used and the electrodes were placed on the left side of the patient’s forehead in accordance with the manufacturer’s instructions. The SctO_2_ was monitored using a FORE-SIGHT Cerebral Oximeter (CAS Medical Systems, Branford, CT, USA). The NIRS pads were placed on the right side of the patient’s forehead directly over the eyebrow and the signal was adjusted to a full signal state [27] (Supplementary Figure S1).

Anesthesia was induced with midazolam 0.15 mg kg^−1^, etomidate 0.3 mg kg^−1^, sufentanil 0.5 µg kg^−1^ and cisatracurium 0.15 mg kg^−1^, followed by endotracheal intubation. Anesthesia was then maintained using propofol 100–200 µg kg^−1^ min^−1^ and remifentanil 0.05–0.25 µg kg^−1^ min^−1^. The rate of propofol administration during maintenance of anesthesia was adjusted to keep the BIS value between 40–60. To minimize the influence of residual paralysis on the evaluation of anesthesia recovery during the maintenance period, no muscle relaxants were used or the last injection of muscle relaxants was more than one hour before the end of the operation, provided that the anesthesia management has reached clinical needs.

At the end of the surgery, the delivery of anesthetics was stopped. The mechanical ventilation was kept at a fraction of inspiration oxygen (FiO_2_) of 30%, and the ventilation parameters were adjusted to maintain the SpO_2_ at 95–100% and the end-tidal carbon dioxide (EtCO_2_) at 35–40 mmHg. The patients were carefully guarded without intentional disturbance until they showed spontaneously appearing behavioral signs, such as body movement, coughing and eye opening [28,29,30]. Once the behavioral signs were identified, the patients were tested to determine whether they regained consciousness or not. The regaining of consciousness was defined if the patients was arousable and able to respond to commands, including directed eye movements and hand shaking. The test was repeated at a 2 min interval until the patient regained consciousness. The patients were given neostigmine 0.04 mg kg^−1^ plus atropine 0.01 mg kg^−1^ to reverse residual neuromuscular block. The extubation was performed when the patients maintained EtCO_2_ < 45 mm Hg and SpO_2_ > 95% with spontaneous breathing room air.

The emergence period was defined as the time from the cessation of anesthetic delivery until the patient regained consciousness. At the beginning of emergence, the SctO_2_, BIS, MAP and HR values were recorded as baseline values. They were continuously recorded thereafter at a 2 min interval during the emergence period. The changes of these parameters over the baseline values were recorded as Δ SctO_2_, Δ BIS, Δ MAP, and Δ HR, as we described above. The Δ SctO_2_, Δ BIS, Δ MAP, and Δ HR were compared at the following time-points during anesthesia emergence, 2 min before the appearance of behavioral signs, appearance of behavioral signs and regaining of consciousness.

### 2.4. Statistical Analysis

Based on the results of our previous observations, the difference of SctO_2_ between “2 min before appearance of behavioral signs” and “Appearance of behavioral signs” to detect was 2.2, with a standard deviation of 7.5 in the “Appearance of behavioral signs” and an autocorrelation of 0.665. Therefore, a sample size of 190 was required with power of 90%, and a significance level of 0.05. Taking into account the possible 5% dropout rate, the total sample size required was 200. The “Test for Two Means in a Repeated Measures Design” mode of PASS 11 (NCSS, LIc., Kaysville, UT, USA) was used to perform these calculations.

Data were presented as mean ± SD (standard deviation) or numbers and percentages (%). All statistical analyses were conducted using SPSS 18.0 (SPSS Inc., Chicago, IL, USA) and GraphPad Prism 7.0 (GraphPad Software Inc., San Diego, CA, USA). Shapiro–Wilk test was used for evaluation of data distribution. To compare normally distributed variables between the two groups, independent *t*-test was used if their variances were equal (using Levene’s test to assess the equality of variances), or Welch’s *t*-test was used if their variances were not equal. To compare non-normally distributed variables between the two groups, Mann–Whitney U test was used. To compare variables between the two time points within one group of patients, paired *t*-test was used if the variables were normally distributed, and Wilcoxon matched pairs signed rank test was used if the variables were not normally distributed.

The accuracy of Δ SctO_2_, Δ BIS, Δ MAP, and Δ HR to predict the appearance of behavioral signs (“appearance of behavioral signs” versus “2 min before appearance of behavioral signs”) was analyzed with the prediction probability (Pk). Pk was calculated for all parameters using a custom spreadsheet macro, PKMACRO, as previously described [31]. A paired t-test was used for the comparison between Pk values of two monitors. A Pk value of 1 means that the value of the predicting variable always correctly predicts the variable to be predicted. A Pk value of 0.5 means that the indicator prediction is no better than chance alone. Pk and its standard error were estimated with the jack-knife method, based on the assumption that all assessments were independent. A receiver operating characteristic (ROC) curve and the associated areas under the curves (AUC) were generated to characterize the sensitivity and specificity of Δ SctO_2_, Δ BIS, Δ MAP, and Δ HR in detecting the appearance of behavioral signs. The comparison between the AUC of ROC curves was performed by the method of DeLong test [32] using MedCalc v. 10.4.7.0 software (MedCalc Software bvba, Mariakerke, Belgium). A *p* value < 0.05 was considered statistically significant.

## 3. Results

### 3.1. Study Population

**Figure 1:**
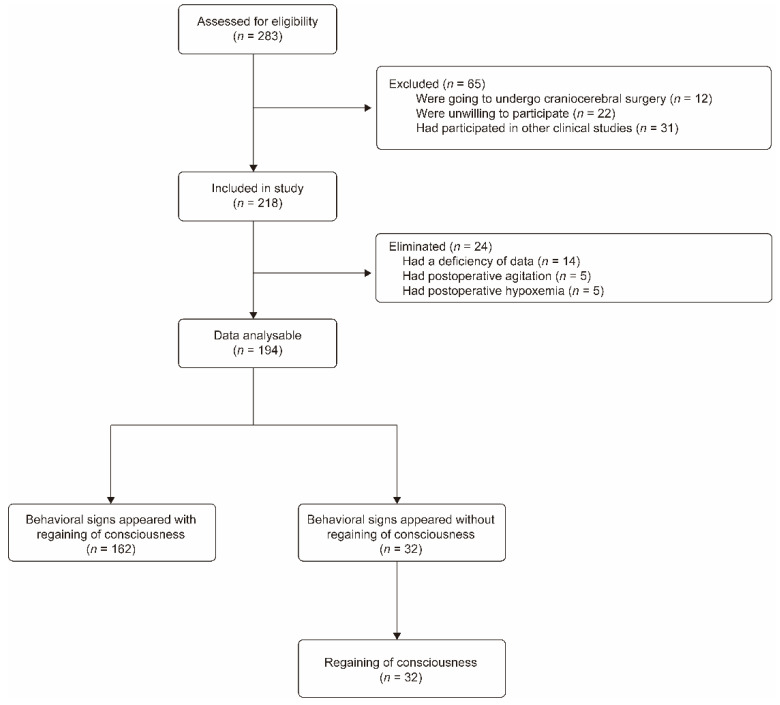
Flow chart of participants’ screening and recruitment.

A total of 218 patients were enrolled in this study. A total of 24 patients among them were eliminated due to data missing (14 patients) or due to hypoxemia or agitation during the period of emergence (10 patients). Eventually, 194 patients completed the study; in addition, 162 of them regained consciousness as soon as the behavioral signs appeared, and the other 32 patients regained consciousness later (Figure 1). The demographic characteristics, types of surgery, intraoperative medications and duration of anesthesia of the patients are shown in Table 1. During the anesthesia emergence, there were no consumption of sedatives, central stimulants, and vasoactive medications.

### 3.2. Appearing of Behavioral Signs during Emergence Is Associated with an Abrupt and Distinctive Increase in SctO_2_ Value

At the beginning of anesthesia emergence, the baseline value of SctO_2_ was 70 ± 6% and it remained stable during the early stage of anesthesia emergence before the behavioral signs appeared. The Δ SctO_2_ at 2 min before behavioral signs appeared was 0 ± 1%. At the moment of the appearance of behavioral signs, the Δ SctO_2_ was 6 ± 3%, which was significantly higher than 2 min before that (*p* < 0.001), demonstrating an abrupt and distinctive increase in SctO_2_ value within such a short interval (Table 2). Multivariable linear regression analyses showed that there was no association of SctO_2_ with MAP, HR, SpO_2_, or EtCO_2_ (Supplementary Table S1).

The baseline values of BIS, MAP, and HR are shown in Table 2. At the moment when behavioral signs appeared, the Δ BIS, Δ MAP, and Δ HR were higher than the values 2 min before, although with a large inter-individual variability among the patients (*p* < 0.001, Table 2). The Δ SctO_2_ showed no correlation with Δ MAP or Δ HR (Supplementary Figure S2), further demonstrating that the SctO_2_ value was changed independently of the hemodynamic alterations during the emergence.

### 3.3. SctO_2_ Is a Prompt and More Reliable Indicator of Appearing Behavioral Signs during Anesthesia Emergence Than BIS, MAP, and HR

**Figure 2:**
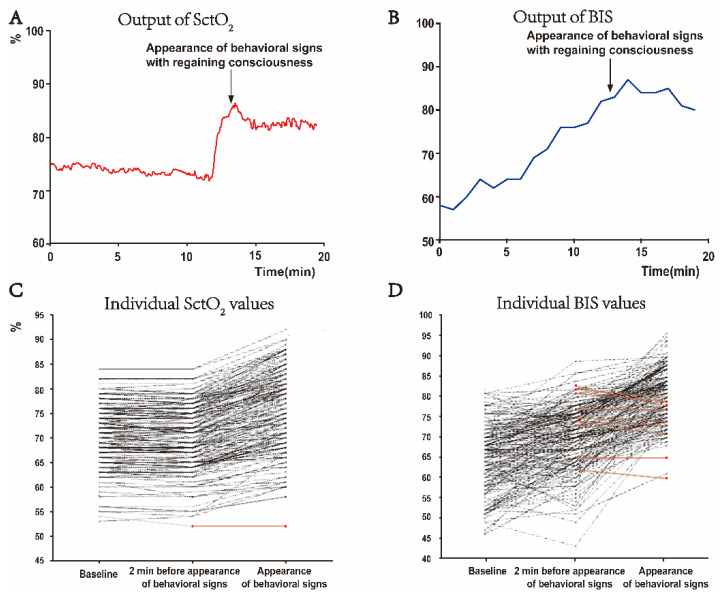
Changes of SctO_2_ and BIS values from baseline to the appearance of behavioral signs. (**A**,**B**) Representative graphs of monitor output of SctO_2_ and BIS. At the moment of appearing behavioral signs, the SctO_2_ value had an obvious peak increase (**A**). The BIS value increased in a relatively stable manner during the emergence period, and there was no special change when the patient had behavioral signs (**B**). (**C**,**D**) Changes of individual SctO_2_ and BIS values (*n* = 194). SctO_2_ value remained relatively stable from the baseline to 2 min before the appearance of behavioral signs, while it was increased in almost every patient when the behavioral signs were appeared (**C**). Changes of BIS value from baseline to the appearance of behavioral signs showed large inter-individual variations (**D**). The black lines represent individual SctO_2_ or BIS values, which were increased at the moment when the behavioral signs appeared compared to 2 min before, while the red lines represent the individual values decreased or unchanged during this interval.

The distinctive increase in SctO_2_ associated with the appearance of behavioral signs was prominent and could easily be identified in the output graph of the SctO_2_ monitor (Figure 2A). In contrast, the increase in BIS value at the appearance of behavioral signs was not particularly different when compared with other time points, since the BIS value rose in a relatively steady pattern during the whole process of anesthesia emergence (Figure 2B). Of the total of 194 patients investigated, 193 of them showed an increase in the SctO_2_ value at the appearance of behavioral signs compared to 2 min before the behavioral signs appeared (Figure 2C), indicating that the increase in SctO_2_ at the moment of the appearance of behavioral signs was a rather universal phenomenon during the emergence from general anesthesia. However, the changes in individual BIS values were not as consistent as SctO_2_ when behavioral signs appeared (Figure 2D). Using Pk analysis to evaluate the ability to predict the appearance of behavioral signs based on the changes of these parameters 2 min before, the Pk score of Δ SctO_2_ was 0.97, which was much higher than Δ BIS (Pk: 0.81), Δ MAP (Pk: 0.72), and Δ HR (Pk: 0.87) (*p* < 0.001, Table 3). The same results were obtained using the ROC analysis and the subsequent DeLong test (Table 3, Supplementary Figure S3). These results demonstrated that SctO_2_ is a prompt and more reliable indicator of anesthesia emergence than BIS, MAP, and HR, within a 2 min interval before behavioral signs appear.

We further investigated the changes of SctO_2_, BIS, MAP and HR in the patients who received a certain type of surgery, including general surgery (*n* = 63), head and neck surgery (*n* = 47) and gynecological surgery (*n* = 25) (Supplementary Table S2), and evaluated the performance of these parameters in predicting anesthesia emergence. The Pk score of Δ SctO_2_ to predict the appearance of behavioral signs was 0.96 in general surgery patients, 0.99 in head and neck surgery patients and 0.97 in gynecological surgery patients, which were much higher than that of Δ BIS, Δ MAP, and Δ HR (*p* < 0.001, Supplementary Table S3). Although we did not evaluate the changes of these parameters in the patients who received other types of surgery due to the small number, our results suggested that the increase in SctO_2_ is a common phenomenon during anesthesia emergence. The SctO_2_ indicated the appearance of behavioral signs regardless of the type of surgery the investigated patients received in our study.

### 3.4. The SctO_2_ Is Further Increased from the Appearance of Behavioral Signs to the Regaining of Consciousness

**Figure 3:**
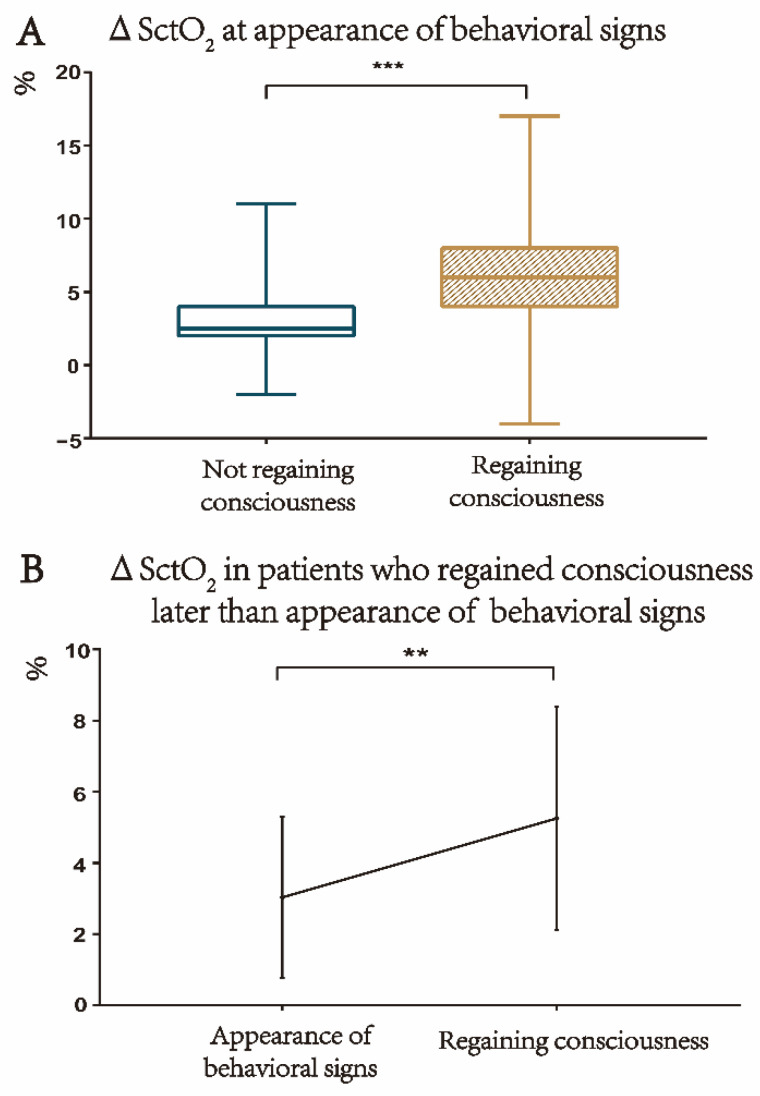
Increase in SctO_2_ value is related to regaining of consciousness. (**A**) At the moment of the appearance of behavioral signs, the change of SctO_2_ over the baseline (Δ SctO_2_) was higher in the patients who also regained consciousness (*n* = 162) than those who did not regain consciousness (*n* = 32), *** *p* < 0.001, Mann–Whitney U test. (**B**) Within the 32 patients who regained consciousness later than the appearance of the behavioral signs, the Δ SctO_2_ was higher at the moment of regaining consciousness than at the moment of behavioral sign appearance, ** *p* < 0.01, paired *t*-test.

The 162 patients who regained consciousness as soon as the behavioral signs appeared and the other 32 patients who did not regain consciousness at the same time showed no differences in their demographics, intraoperative medications or duration of anesthesia (Supplementary Table S4). However, at the moment of the appearance of behavioral signs, the Δ SctO_2_ was higher in the group of the 162 patients who regained consciousness than in the group of the 32 patients who did not regain consciousness (*p* < 0.001, Figure 3A). In these 32 patients, the consciousness returned in 8.25 ± 6.87 min after the onset of behavioral signs. Interestingly, within these patients, the Δ SctO_2_ was higher at the moment of regaining consciousness than at the moment when only the behavioral signs appeared (*p* < 0.01, Figure 3B). Multivariable linear regression analyses showed that the SctO_2_ was not associated with MAP, HR, SpO_2_, or EtCO_2_ (Supplementary Table S1). These results further indicate that the increase in SctO_2_ correlated with the process of emergence.

## 4. Discussion

In this study, we identified an abrupt and distinctive increase in SctO_2_ as soon as the patient showed behavioral signs during the emergence from propofol-remifentanil anesthesia. The BIS, MAP, and HR values were also increased, but with a relatively high inter-individual variability at the appearance of behavioral signs. The measurement of SctO_2_ showed a higher accuracy to predict anesthesia emergence than that of BIS, MAP, and HR, within a 2 min interval prior to the appearance of behavioral signs. The regaining of consciousness was associated with a higher SctO_2_ value than when only behavioral signs appeared, indicating a relationship between the increase in SctO_2_ and the recovery of consciousness after general anesthesia.

SctO_2_ monitoring has been extensively used to provide an index of organ ischemia [20]. This study shows for the first time that SctO_2_ could be an indicator of anesthesia emergence. SctO_2_ remained stable during the early stage of emergence and was not changed until the behavioral signs appeared. The abrupt and distinctive increase in SctO_2_ associated with the appearance of behavioral signs could be easily identified by the anesthesia practitioners via the monitor, and then the assessment for extubation could be conducted timely, thus contributing to early tracheal extubation and less man-machine counteraction. In clinical practice, anesthesiologists tend to use behavioral signs to determine the timing of extubation. However, in some settings, especially when caring for multiple patients awaiting anesthetic awakening and extubation (e.g., in a post-anesthesia care unit), anesthesiologists sometimes do not detect behavioral signs in a timely manner. Therefore, a sudden increase in SctO_2_ can be a more effective indicator of patient awakening because it is more visible than behavioral signs. Moreover, the increase in SctO_2_ during emergence was a common phenomenon and was not influenced by the type of surgery. We further showed that changes in the SctO_2_ value were not related to changes in hemodynamic parameters including MAP and HR. This is consistent with previous reports showing that the emergence-related changes in cerebral circulation were not related to the systemic hemodynamic changes [33]. Taken together, our results suggest that SctO_2_ could be a prompt and reliable indicator of emergence from anesthesia. However, it should be noticed that several factors may influence cerebral oxygen transport and oxygen saturation including hematocrit, inspiratory oxygenation, and ventilation [34,35]. It is essential to maintain a stable concentration of hemoglobin, FiO_2_, SpO_2_, and EtCO_2_ when using the SctO_2_ to assess the emergence from anesthesia.

The BIS, MAP and HR showed patterns of changes which were different from that of SctO_2_ during emergence. BIS values were progressively increased from the beginning of emergence and there was no distinctive change at any state of the emergence period. Moreover, the changes of BIS showed a relatively large inter-individual differences among the patients. Thus, different from the increase in SctO_2_ which indicated the behavioral signs within a 2 min interval, the change of BIS did not rapidly and reliably reflect the transition of emergence state [10,36]. The changes of MAP and HR also showed large individual differences during the emergence, probably due not only to the influence of anesthetics, but also to many other clinical factors that can cause systemic hemodynamic changes [37,38,39].

It has been accepted that anesthesia emergence does not establish at once but in a bottom-up manner [40]. After ceasing anesthetics, there will be a slow return of brainstem reflexes, eventually leading to uncoordinated body movements that occur shortly before subjects regain consciousness [40,41]. We showed that the regaining of consciousness was associated with a higher SctO_2_ value than when only behavioral signs appeared. This result further indicates that the increase in SctO_2_ correlated with the process of emergence. However, the emergence from anesthesia involves a complex interplay of different brain regions that can show different changes in neuronal activity and circulation [17]. Furthermore, it is possible that the NIRS only reflects the SctO_2_ change in the prefrontal cortex [42,43]. Thus, further studies are needed to better understand the details of cerebral oxygen saturation changes during anesthesia emergence.

The following limitations of the present study should be noted. First, the neuromuscular function was not monitored by the train-of-four during the emergence period. In order to minimize the residual effects of muscle relaxant during the emergence, we included the patients who did not receive muscle relaxant during anesthesia maintenance or who received the last injection of muscle relaxant more than one hour before the end of surgery. However, the potential confounding role of muscle relaxants still could not to be ruled out when evaluating the physical and behavioral signs during the emergence. Second, the data of pre-anesthesia induction and during deep anesthesia state were not collected in the present study. Considering that induction and emergence from general anesthesia are not mirror opposite processes [12,44], we focused on the evaluation of emergence process. The baseline of data was set at the beginning of emergence. This might be appropriate for the measurement of SctO_2_ which remained stable during the early period of emergence before the appearance of behavioral signs. However, it should be noted that the depth of anesthesia may vary among patients, which may lead to individual differences in baseline and changes in BIS values. Third, the evaluation in this study was only performed in adult patients. Nevertheless, compared with adult patients, the assessment of pediatric anesthesia recovery relies more on objective measurement, because children are usually uncooperative or even nonverbal. Further experiments should be conducted to evaluate whether SctO_2_ can be used as an indicator of emergence from anesthesia in pediatric patients. Fourth, the patients who received volatile anesthesia were not included in this study. Further studies will be required to compare the SctO_2_ between the emergence from anesthesia maintained by total intravenous anesthesia or volatile agents. Fifth, SctO_2_ monitoring is usually applied in some types of surgery, which have a great impact on cerebral perfusion (e.g., cardiac surgery, carotid endarterectomy). However, most of the surgery types included in this study did not routinely use SctO_2_ monitoring in clinical practice. It may limit the significance of our findings in clinical practice. Despite all this, through this study, SctO_2_, as a non-invasive and well performed monitoring, is potentially another valuable index in the emergence from general anesthesia.

## 5. Conclusions

The increase in SctO_2_ correlated with the emergence from propofol-remifentanil anesthesia. SctO_2_ is a more reliable indicator of appearing behavioral signs during anesthesia emergence than BIS, MAP, and HR, within a 2 min interval prior to the appearance of behavioral signs.

## Figures and Tables

**Table 1:** Patients’ demographic characteristics, types of surgery, intraoperative medications, and duration of anesthesia.

Characteristics	Patients (*n* = 194)
Age (y)	49.41 ± 12.39
Male, *n* (%)	91 (46.91)
BMI (kg (m^−2^)^−1^)	23.03 ± 2.86
ASA classification, *n* (%)	
II	120 (61.86)
III	74 (38.14)
Comorbidities, *n* (%)	
Hypertension	55 (28.35)
Current smoker	54 (27.84)
Diabetes	29 (14.95)
Coronary artery disease	24 (12.37)
Asthma	21 (10.82)
Chronic obstructive pulmonary disease	12 (6.19)
Obesity ^a^	5 (2.58)
Type of surgery, *n* (%)	
Head and neck	47 (24.23)
General	63 (32.47)
Gynecological	25 (12.89)
Thoracic	18 (9.28)
Orthopedic	8 (4.12)
Spinal	7 (3.61)
Vascular	7 (3.61)
Plastic	5 (2.58)
Other	14 (7.21)
Intraoperative medications	
Midazolam (mg)	7.20 ± 1.99
Sufentanil (µg)	36.88 ± 9.55
Cisatracurium (mg)	16.62 ± 3.80
Etomidate (mg)	22.40 ± 14.60
Propofol (mg kg^−1^)	17.75 ± 9.44
Remifentanil (µg kg^−1^)	25.88 ± 13.84
Duration of anesthesia (min)	134.39 ± 67.20

Values are mean ± SD or numbers and percentages (%). BMI, body mass index; ASA, American Society of Anesthesiologists. ^a^ Defined as body mass index greater than 30.

**Table 2:** Physiological values from the beginning of emergence to the appearance of behavioral signs.

	Baseline *	Changes over Baseline ^#^		
		2 Min before Appearance of Behavioral Signs ^&^	Appearance of Behavioral Signs	*p* Values ^$^
SctO_2_ (%)	70 ± 6	0 ± 1	6 ± 3	<0.001
BIS	65 ± 8	6 ± 6	16 ± 9	<0.001
MAP (mmHg)	89 ± 13	1 ± 5	5 ± 7	<0.001
HR (bpm)	60 ± 10	1 ± 5	13 ± 10	<0.001

Data are mean ± SD. SctO_2_, cerebral tissue oxygen saturation; BIS, bispectral index; MAP, mean arterial pressure; HR, heart rate. * “Baseline” refers to the values of SctO_2_, BIS, MAP and HR recorded at the beginning of emergence. ^#^ “Changes over baseline” refers to the difference between the values of SctO_2_, BIS, MAP and HR at 2 min before the appearance of behavioral signs or at the moment of appearance of behavioral signs and the baseline values of each variable. ^&^ “Behavioral signs” refers to the first appearance of behavioral signs indicating emergence, including body movement, coughing or eye opening. ^$^ The value changes of SctO_2_, BIS, MAP and HR at “2 min before appearance of behavioral signs” versus “appearance of behavioral signs”, *p* < 0.001, using Wilcoxon matched-pairs signed rank test.

**Table 3:** Prediction performance of the four parameters for the appearance of behavioral signs.

	Pk	SE	AUC	95% CI
Δ SctO_2_	0.97	0.01	0.97	0.95–0.99
Δ BIS	0.81 ***	0.02	0.81 ^###^	0.77–0.85
Δ MAP	0.72 ***	0.03	0.72 ^###^	0.67–0.76
Δ HR	0.87 ***	0.02	0.87 ^###^	0.83–0.90

Pk, prediction probability; SE, standard error; AUC, the associated areas under the receiver operating characteristic (ROC) curves; CI, confidence interval. Δ SctO_2_, Δ BIS, Δ MAP, and Δ HR refer to the changes of SctO_2_, BIS, MAP, and HR values over the baseline value of each parameter. The accuracy of Δ SctO_2_ to predict the appearance of behavioral signs (“appearance of behavioral signs” versus “2 min before appearance of behavioral signs”) was higher than that of Δ BIS, Δ MAP, and Δ HR, *** *p* < 0.001, Pk analysis followed by paired *t*-test; ^###^ *p* < 0.001, ROC analysis followed by DeLong test.

## Data Availability

All data included in this study are available upon request by contact with the corresponding author.

## Supplementary Materials

The following supporting information can be downloaded at: https://www.mdpi.com/article/10.3390/jcm11164878/s1, Figure S1: The placement of BIS and NIRS. Figure S2: There are no correlations between Δ SctO_2_ and Δ MAP or Δ HR at appearance of behavioral signs. (A) Δ SctO_2_ does not correlate with Δ MAP (r = 0.1518, *p* = 0.0346). (B) Δ SctO_2_ weakly correlates with Δ HR (r = 0.2159, *p* = 0.0025). Figure S3: Performance of Δ SctO_2_, Δ BIS, Δ MAP and Δ HR in predicting the appearance of behavioral signs using the receiver operating characteristic (ROC) curves, *n* = 194. (A) ROC of Δ SctO_2_, Δ BIS, Δ MAP and Δ HR for predicting the appearance of behavioral signs. (B,C) Comparison of diagnostic accuracy among Δ SctO_2_, Δ BIS, Δ MAP and Δ HR for predicting the appearance of behavioral signs using the diagnostic parameters. Table S1: The multivariate analysis of SctO_2_ at with other parameters (EtCO_2_, SpO_2_, MAP and HR), *n* = 194. Table S2: Physiological values from the beginning of emergence to the appearance of behavioral signs in patients receiving different types of surgery. Table S3: Performance of Δ SctO_2_, Δ BIS, Δ MAP and Δ HR in predicting appearance of behavioral signs in patients receiving different types of surgeries. Table S4: Main characteristics of patients who regained consciousness when behavioral signs appeared (*n* = 162) and those who regained consciousness later than the appearance of behavioral signs (*n* = 32).
