# Supplementary material for: Cerebral Tissue Oxygen Saturation Correlates with Emergence from Propofol-Remifentanil Anesthesia: An Observational Cohort Study
Source: J Clin Med. 2022 Aug 19;11(16):4878. doi: 10.3390/jcm11164878 (PMC9410034; doi:10.3390/jcm11164878)
Supplement: Supplementary file 1 [file jcm-11-04878-s001.zip › jcm-1801044-supplementary.pdf]

Supplemental materials

Cerebral tissue oxygen saturation correlates with emergence from propofol-remifentanil anesthesia: an observational cohort study

Jianxi Zhang, Zhigang Cheng, Ying Tian, Lili Weng, Yiying Zhang, Xin Yang, Michael K. E. Schäfer, Qulian Guo, and Changsheng Huang

1. **Methods**

Data were presented as mean ± SD (standard deviation) or numbers and percentages (%). All statistical analyses were conducted using SPSS 18.0 (SPSS Inc., Chicago, Illinois, USA) and GraphPad Prism 7.0 (GraphPad Software Inc., San Diego, California, USA). Shapiro-Wilk test was used for evaluation of normality. To compare normally distributed variables between the two groups, independent t-test was used if their variances were equal (using Levene's test to assess the equality of variances), or Welch's t-test was used if their variances were not equal. To compare non-normally distributed variables between the two groups, Mann–Whitney U test was used. To compare variables between the two time points within one group of patients, paired t-test was used if the variables were normally distributed, and Wilcoxon matched pairs signed rank test was used if the variables were not normally distributed. To compare the categorical variables, chi-square test and Fisher exact tests were used. Pearson's correlation analysis was conducted to examine the association between Δ $SctO_2$ and Δ MAP or Δ HR at the appearance of behavioral signs. Multivariable linear regression analyses were performed to examine the associations of $SctO_2$ at different time points with MAP, HR, SpO_2 and $EtCO_2$.

The accuracy of Δ $SctO_2$, Δ BIS, Δ MAP and Δ HR to predict the appearance of behavioral signs ("appearance of behavioral signs" versus "2 min before appearance of behavioral signs") was analyzed by the prediction probability (Pk). Pk was calculated for all parameters using a custom spreadsheet macro, PKMACRO. A Pk value of 1 means that the value of the predicting variable always correctly predicts the variable to

be predicted. A Pk value of 0.5 means that the indicator prediction is not better than by chance alone. Pk and its standard error were estimated with the jack-knife method, based on the assumption that all assessments were independent. A paired t-test and Bonferroni correction were used for the comparison between Pk values of two monitors.

Additionally, a receiver operating characteristic (ROC) curve and the associated areas under the curves (AUC) were generated to characterize the sensitivity and specificity of Δ $SctO_2$, Δ BIS, Δ MAP and Δ HR in detecting the appearance of behavioral signs. The comparison between the AUC of ROC curves was performed by the method of DeLong test using MedCalc v. 10.4.7.0 software (MedCalc Software bvba, Mariakerke, Belgium). A P value <0.05 was considered statistically significant.

2. Results

The BIS VISTA monitor (Aspect Medical Systems, Newton, MA) was used and the electrodes were placed on the left side of the patient's forehead in accordance with the manufacturer's instructions. The $SctO_2$ was monitored using a FORE-SIGHT Cerebral Oximeter (CAS Medical Systems, Branford CT USA). The NIRS pads were placed on the right side of the patient's forehead directly over the eyebrow and the signal was adjusted to a full signal state (**Supplemental Figure S1**).

Multivariable linear regression analyses showed that the $SctO_2$ had no association with MAP, HR, SpO_2 or $EtCO_2$ at different time points (beginning of anesthesia emergence, 2 min before appearance of behavioral signs, appearance of behavioral signs and regaining of consciousness) (**Supplemental Table S1**).

Pearson's correlation analysis showed that there are no correlations between Δ $SctO_2$ and Δ MAP or Δ HR at the appearance of behavioral signs (**Supplemental Figure S2**).

The sensitivity and specificity in ROC curves of the Δ $SctO_2$ (Sensitivity = 92.27 %, Specificity = 94.85 %, AUC = 0.97, 95% CI: 0.95–0.99) for detecting the appearance of behavioral signs were high compared to Δ BIS (Sensitivity = 59.28 %, Specificity = 89.69 %, AUC = 0.81, 95% CI: 0.77–0.85), Δ MAP (Sensitivity = 62.37 %, Specificity = 75.77 %, AUC = 0.72, 95% CI: 0.67–0.76) and Δ HR (Sensitivity = 78.87 %, Specificity = 89.18 %, AUC = 0.87, 95% CI: 0.83–0.90) ($P < 0.001$) (**Supplemental Figure S3**).

We investigated the changes of $SctO_2$, BIS, MAP and HR in the patients who received a certain type of surgery, including general surgery (n = 63), head and neck surgery (n = 47) and gynecological surgery (n = 25) (**Supplemental Table S2**), and evaluated the performance of these parameters in predicting anesthesia emergence. The Pk score of Δ $SctO_2$ to predict the appearance of behavioral signs was 0.96 in general surgery patients, 0.99 in head and neck surgery patients, and 0.97 in gynecological surgery patients, which were much higher than that of Δ BIS, Δ MAP and Δ HR ($P < 0.001$, **supplemental Table S3**). Although we did not evaluate the changes of these parameters in the patients who received other types of surgery due to their small number, our results indicated that the increase of $SctO_2$ is a common phenomenon during anesthesia emergence. The $SctO_2$ indicated the appearance of behavioral signs independent of the types of surgery investigated in our study.

Among the 194 patients investigated, 162 of them regained consciousness as soon as the behavioral signs appeared. In contrast, the other 32 patients did not return consciousness when behavioral signs appeared. These two groups of patients showed no differences with in demographic characteristics, intraoperative medications or duration of anesthesia (**Supplemental Table S4**).

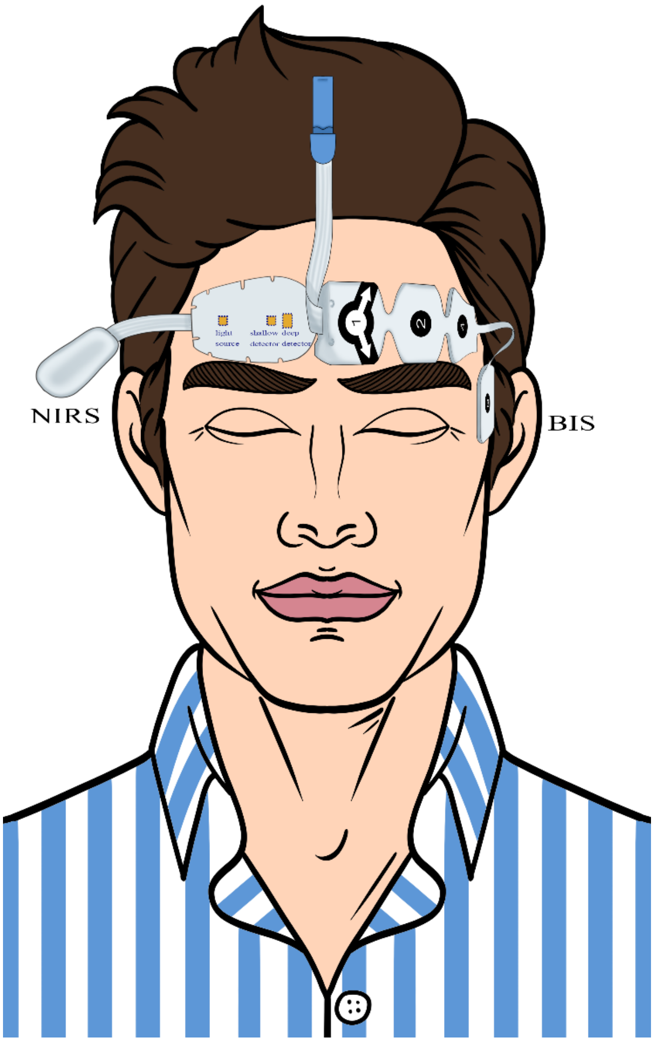

Supplemental Figure S1 The placement of BIS and NIRS. BIS, Bispectral Index; NIRS, near-infrared spectroscopy.

Supplemental Table S1. The multivariate analysis of $SctO_2$ at with other parameters ($EtCO_2$, SpO_2, MAP and HR), n = 194.

	$SctO_2$ at the beginning of anesthesia emergence		$SctO_2$ at 2 min before appearance of behavioral signs	
	Effect estimate (95% CI)	P value	Effect estimate (95% CI)	P value
$EtCO_2$	0.46 (-0.11, 1.03)	0.11	0.44 (-0.08, 0.95)	0.09
SpO_2	-1.06 (-2.24, 0.11)	0.08	-0.78 (-1.94, 0.38)	0.19
HR	-0.02 (-0.11, 0.07)	0.73	-0.02 (-0.11, 0.07)	0.65
MAP	-0.02 (-0.09, 0.05)	0.63	-0.02 (-0.10, 0.05)	0.51

	$SctO_2$ at the appearance of behavioral signs		$SctO_2$ at the regaining of consciousness	
	Effect estimate (95% CI)	P value	Effect estimate (95% CI)	P value
$EtCO_2$	0.64 (-0.05, 1.32)	0.07	1.10 (-0.86, 3.06)	0.26
SpO_2	-0.63 (-2.01, 0.76)	0.37	-2.32 (-8.54, 3.90)	0.45
HR	0.05 (-0.05, 0.15)	0.31	0.03 (-0.23, 0.28)	0.84
MAP	0.01 (-0.09, 0.11)	0.85	0.10 (-0.11, 0.30)	0.99

$SctO_2$, cerebral tissue oxygen saturation; $EtCO_2$, end-tidal carbon dioxide tension; SpO_2, pulse oxygen saturation; MAP, mean arterial pressure; HR, heart rate.

Effect size are presented as betas and should be interpreted as follows: an increase of one unit of the covariate will increase the $SctO_2$ by beta times.

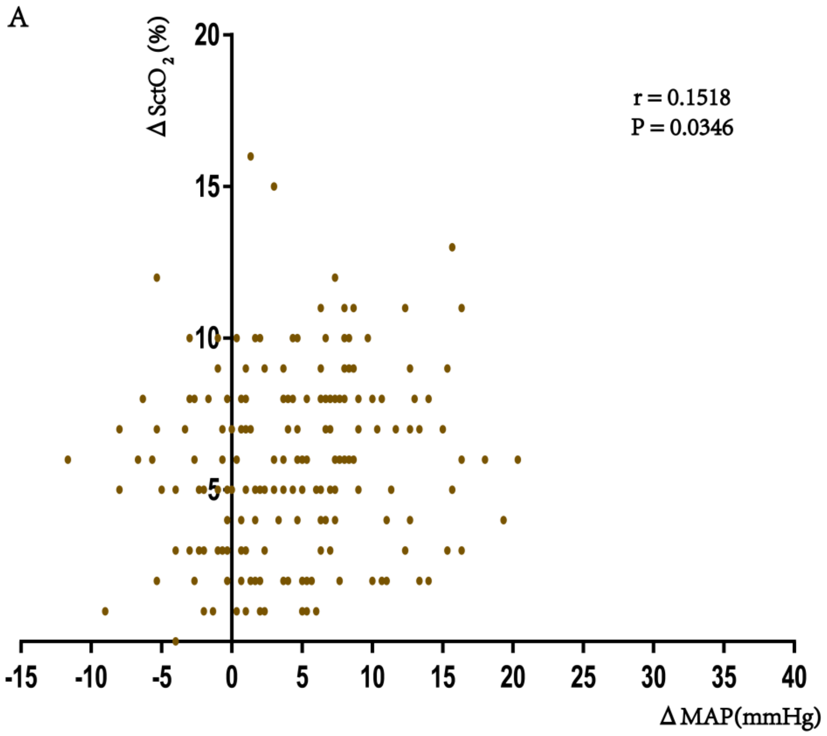

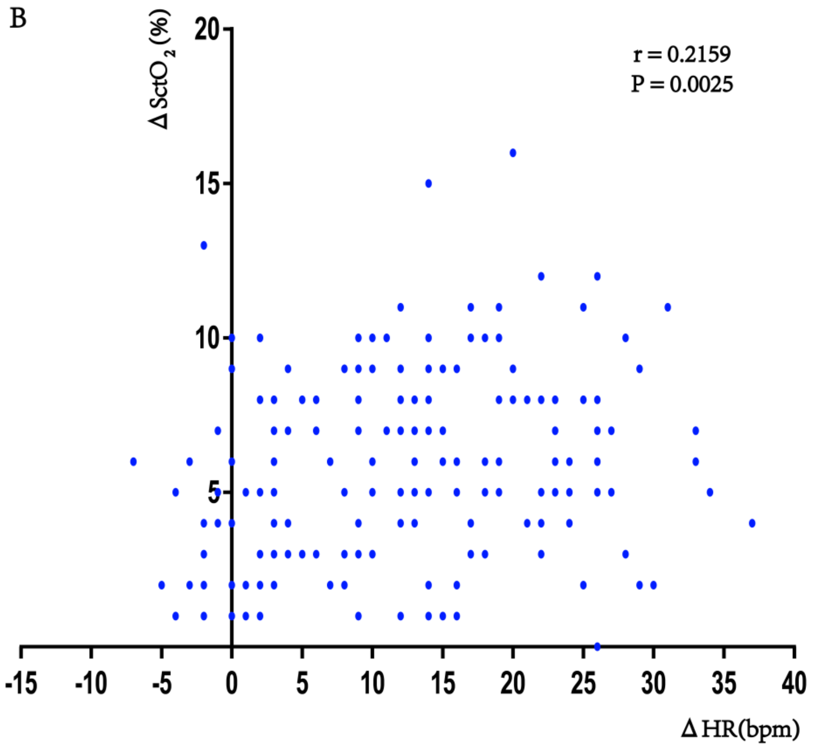

Supplemental Figure S2 There are no correlations between Δ SctO_2 and Δ MAP or Δ HR at appearance of behavioral signs. (A) Δ SctO_2 does not correlate with Δ MAP (r = 0.1518, p = 0.0346). (B) Δ SctO_2 weakly correlates with Δ HR (r = 0.2159, p = 0.0025).

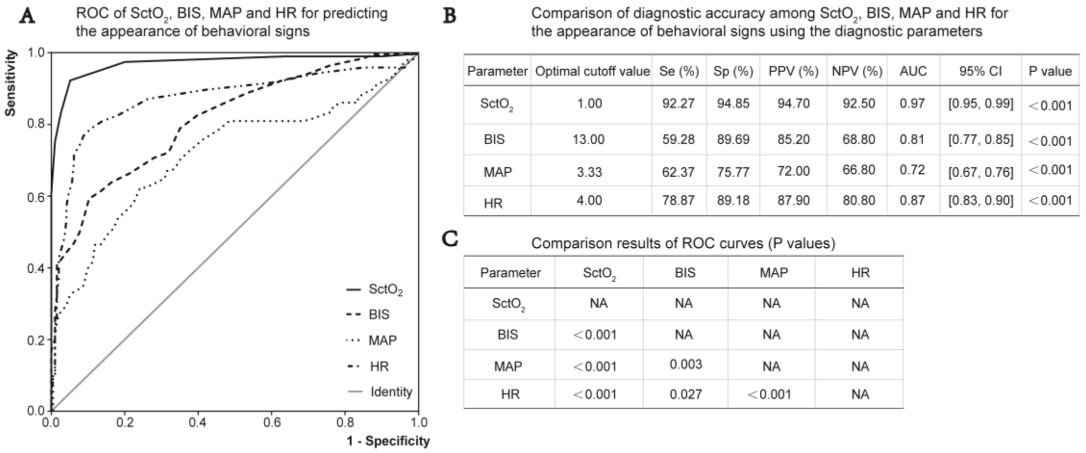

Parameter	Optimal cutoff value	Se (%)	Sp (%)	PPV (%)	NPV (%)	AUC	95% CI	P value
$SctO_2$	1.00	92.27	94.85	94.70	92.50	0.97	[0.95, 0.99]	< 0.001
BIS	13.00	59.28	89.69	85.20	68.80	0.81	[0.77, 0.85]	< 0.001
MAP	3.33	62.37	75.77	72.00	66.80	0.72	[0.67, 0.76]	< 0.001
HR	4.00	78.87	89.18	87.90	80.80	0.87	[0.83, 0.90]	< 0.001

Parameter	$SctO_2$	BIS	MAP	HR
$SctO_2$	NA	NA	NA	NA
BIS	< 0.001	NA	NA	NA
MAP	< 0.001	0.003	NA	NA
HR	< 0.001	0.027	< 0.001	NA

Supplemental Figure S3 Performance of Δ $SctO_2$, Δ BIS, Δ MAP and Δ HR in predicting the appearance of behavioral signs using the receiver operating characteristic (ROC) curves, n = 194. (A) ROC of Δ $SctO_2$, Δ BIS, Δ MAP and Δ HR for predicting the appearance of behavioral signs. (B-C) Comparison of diagnostic accuracy among Δ $SctO_2$, Δ BIS, Δ MAP and Δ HR for predicting the appearance of behavioral signs using the diagnostic parameters. $SctO_2$, cerebral tissue oxygen saturation; BIS, bispectral index; MAP, mean arterial pressure; HR, heart rate; Se, sensitivity; Sp, specificity; PPV, positive predictive value; NPV, negative predictive value; AUC, the associated areas under the receiver operating characteristic (ROC) curves; 95 % CI, 95% confidence interval.

Supplemental Table S2 Physiological values from the beginning of emergence to the appearance of behavioral signs in patients receiving different types of surgery.

	General surgery (n = 63)			
	Baseline *	Changes over baseline #		P value $
		2 min before appearance of behavioral signs	Appearance of behavioral signs &	

**SctO2（%）**	70 ± 6	0 ± 1	5 ± 3	<0.001
**BIS**	66 ± 7	6 ± 6	14 ± 9	<0.001
**MAP（mmHg）**	93 ± 14	1 ± 5	4 ± 6	<0.001
**HR（bpm）**	60 ± 9	1 ± 5	12 ± 10	<0.001

	**Head and neck surgery (n = 47)**			
	**Baseline** *	**Changes over baseline** #		**P value** $
		**2 min before appearance of behavioral signs**	**Appearance of behavioral signs** &	
**SctO2（%）**	74 ± 6	0 ± 1	6 ± 3	<0.001
**BIS**	65 ± 8	5 ± 7	17 ± 9	<0.001
**MAP（mmHg）**	85 ± 11	1 ± 5	7 ± 6	<0.001
**HR（bpm）**	61 ± 8	1 ± 6	14 ± 9	<0.001

	**Gynecological surgery (n = 25)**			
	**Baseline** *	**Changes over baseline** #		**P value** $
		**2 min before appearance of behavioral signs**	**Appearance of behavioral signs** &	
**SctO2（%）**	69 ± 6	0 ± 1	6 ± 4	<0.001
**BIS**	66 ± 8	5 ± 6	16 ± 9	<0.001
**MAP（mmHg）**	88 ± 15	0 ± 6	3 ± 9	<0.05
**HR（bpm）**	58 ± 11	0 ± 3	14 ± 11	<0.001

Data are mean ± SD.

$SctO_2$, cerebral tissue oxygen saturation; BIS, bispectral index; MAP, mean arterial

pressure; HR, heart rate.

* "Baseline" refers to the values of $SctO_2$, BIS, MAP and HR recorded at the beginning of emergence.

# "Changes over baseline" refers to the difference between the values of $SctO_2$, BIS, MAP and HR at 2 min before the appearance of behavioral signs or at the moment of appearance of behavioral signs and the baseline values of each variable.

& "Behavioral signs" refers to the first appeared behavioral signs indicating emergence, including body movement, coughing or eye opening.

$ The value changes of $SctO_2$, BIS, MAP and HR at "2 min before appearance of behavioral signs" versus "appearance of behavioral sings", P <0.001 or P <0.05, using Wilcoxon matched-pairs signed rank test.

Supplemental Table S3 Performance of Δ $SctO_2$, Δ BIS, Δ MAP and Δ HR in predicting appearance of behavioral signs in patients receiving different types of surgeries.

**General (n = 63)**				
	Pk	SE	AUC	95% CI
Δ $SctO_2$	0.96	0.02	0.96	0.91-0.99
Δ BIS	0.78***	0.04	0.78###	0.70-0.85
Δ MAP	0.68***	0.05	0.68###	0.59-0.76
Δ HR	0.86***	0.04	0.86#	0.79-0.92

**Head and neck (n = 47)**				
	Pk	SE	AUC	95% CI

Δ $SctO_2$	0.99	0.01	0.99	0.94-1.00
Δ BIS	0.86^{***}	0.04	$0.86^{\#\#\#}$	0.77-0.92
Δ MAP	0.80^{***}	0.05	$0.80^{\#\#\#}$	0.71-0.88
Δ HR	0.91^{***}	0.03	$0.91^{\#}$	0.84-0.96

**Gynecological (n = 25)**				
	Pk	SE	AUC	95% CI
Δ $SctO_2$	0.97	0.02	0.97	0.87-1.00
Δ BIS	0.82^{***}	0.06	$0.82^{\#}$	0.69-0.92
Δ MAP	0.62^{***}	0.09	$0.63^{\#\#\#}$	0.48-0.76
Δ HR	0.88^{***}	0.06	0.88	0.76-0.95

Pk, prediction probability; SE, standard error; AUC, the associated areas under the receiver operating characteristic (ROC) curves; 95 % CI, 95% confidence interval.

Δ $SctO_2$, Δ BIS, Δ MAP and Δ HR refer to the changes of $SctO_2$, BIS, MAP and HR values over the baseline value of each parameter.

The accuracy of Δ $SctO_2$ to predict appearance of behavioral signs ("appearance of behavioral signs" versus "2 min before appearance of behavioral signs") was higher than that of Δ BIS, Δ MAP and Δ HR, $^{***}P < 0.001$, paired t-test.

The sensitivity and specificity in ROC curves of Δ $SctO_2$ to predict appearance of behavioral signs ("appearance of behavioral signs" versus "2 min before appearance of behavioral signs") was higher than that of Δ BIS, Δ MAP and Δ HR. $^{\#\#\#}P < 0.001$, $^{\#}P < 0.05$, DeLong test.

Supplemental Table S4 Main characteristics of patients who regained consciousness when behavioral signs appeared (n = 162) and those who regained consciousness later than the appearance of behavioral signs (n = 32).

	Regaining consciousness when behavioral signs appeared (n = 162)	Regaining consciousness later than the appearance of behavioral signs (n = 32)	P-value
Age (yr), mean±SD	49.81±12.37	47.38±12.41	0.21[a]
Male, n (%)	79(48.77)	12(37.50)	0.24[b]
BMI (kg/m^2), mean±SD	23.07±2.97	22.96±2.71	0.62[a]
ASA classification, n (%)			0.93[b]
II	100(61.73)	20(62.50)	
III	62(38.27)	12(37.50)	
Duration of anesthesia (min), mean±SD	131.34±67.31	149.81±66.57	0.10[a]
Intraoperative medications			
Midazolam (mg)	7.12±2.04	7.64±1.70	0.13[a]
Sufentanil (ug)	36.50±9.99	38.81±6.69	0.11[a]
Cisatracurium (mg)	16.59±5.79	16.78±3.91	0.62[a]
Etomidate (mg)	21.35±3.20	21.50±2.78	0.91[a]
Profofol ($mg\ kg^{-1}$)	17.23±9.17	20.38±10.51	0.12[a]

Remifentanil (ug kg $^{-1}$)	25.11±13.40	29.75±15.51	0.12[a]

Values are mean ± SD or numbers and percentages (%).

BMI, body mass index; ASA, American Society of Anesthesiologists

[a] Main characteristics like age, BMI, duration of anesthesia and intraoperative medications in the patients “Regaining consciousness when behavioral signs appeared” versus “Regaining consciousness later than the appearance of behavioral signs”, P > 0.05, using Mann–Whitney U test.

[b] The comparison of “Male” and “ASA classification” in the patients “Regaining consciousness when behavioral signs appeared” versus “Regaining consciousness later than the appearance of behavioral signs”, P > 0.05, using chi-square test
